# Echocardiographic Diagnosis of Ventricular Free Wall Rupture Complicating Takotsubo Syndrome: A Case Report

**DOI:** 10.7759/cureus.107382

**Published:** 2026-04-20

**Authors:** Akihiro Mizuno

**Affiliations:** 1 Department of Cardiology, Eiju General Hospital, Tokyo, JPN

**Keywords:** apical ballooning, cardiac tamponade, echocardiography, ischemic stroke, stress cardiomyopathy, takotsubo syndrome, ventricular free wall rupture

## Abstract

Takotsubo syndrome (TTS), also known as Takotsubo cardiomyopathy, is generally considered a reversible condition; however, it can be associated with fatal complications. We report a case of ventricular free wall rupture diagnosed by echocardiography in TTS triggered by an acute ischemic stroke. An 84-year-old man presented with left hemiparesis and was diagnosed with an acute right parietal infarction. On hospital day six, electrocardiography revealed diffuse ST-segment elevation without chest pain. Transthoracic echocardiography demonstrated akinesis of the mid-to-apical segments of the left ventricle with compensatory hyperkinesis of the basal segments, resulting in a typical apical ballooning pattern. These findings were inconsistent with a single coronary artery territory and supported the diagnosis of TTS. Coronary angiography revealed moderate coronary stenoses; however, left ventriculography demonstrated wall motion abnormalities that did not correspond to a single coronary distribution, further supporting the diagnosis of TTS. Several hours later, the patient developed sudden cardiac arrest. Transthoracic echocardiography revealed pericardial effusion with fibrin-like echoes, suggestive of ventricular free wall rupture. Despite resuscitative efforts, the patient died.

## Introduction

Takotsubo syndrome (TTS), also known as stress-induced cardiomyopathy, is characterized by transient left ventricular systolic dysfunction, typically triggered by physical or emotional stress [1,2]. Diagnostic criteria generally include transient regional wall motion abnormalities extending beyond a single coronary artery territory, absence of obstructive coronary artery disease or plaque rupture, new electrocardiographic abnormalities, and modest elevation of cardiac biomarkers.

Although TTS was historically regarded as a benign and reversible condition, recent evidence has demonstrated that it is associated with a substantial risk of complications. Data from large registries indicate that acute complications occur in approximately 20% of patients, a rate comparable to acute coronary syndrome (ACS) [3,4]. These complications include cardiogenic shock, ventricular arrhythmias, thromboembolism, and, in rare cases, cardiac rupture.

Neurological disorders such as ischemic stroke are well-recognized triggers of TTS. The underlying pathophysiology is thought to involve excessive activation of the sympathetic nervous system and catecholamine surge, resulting in myocardial stunning. The International Takotsubo Registry has shown that neurological and psychiatric conditions are present in a substantial proportion of TTS cases and are associated with worse outcomes [3].

Cardiac rupture is an extremely rare but catastrophic complication of TTS, with reported incidence generally below 1%, yet it carries an extremely high mortality rate [5].

Here, we report a rare case of TTS triggered by an acute ischemic stroke that resulted in ventricular free wall rupture. This case highlights the diagnostic value of echocardiography in detecting this fatal complication in a time-sensitive clinical setting.

## Case presentation

**Figure 1:**
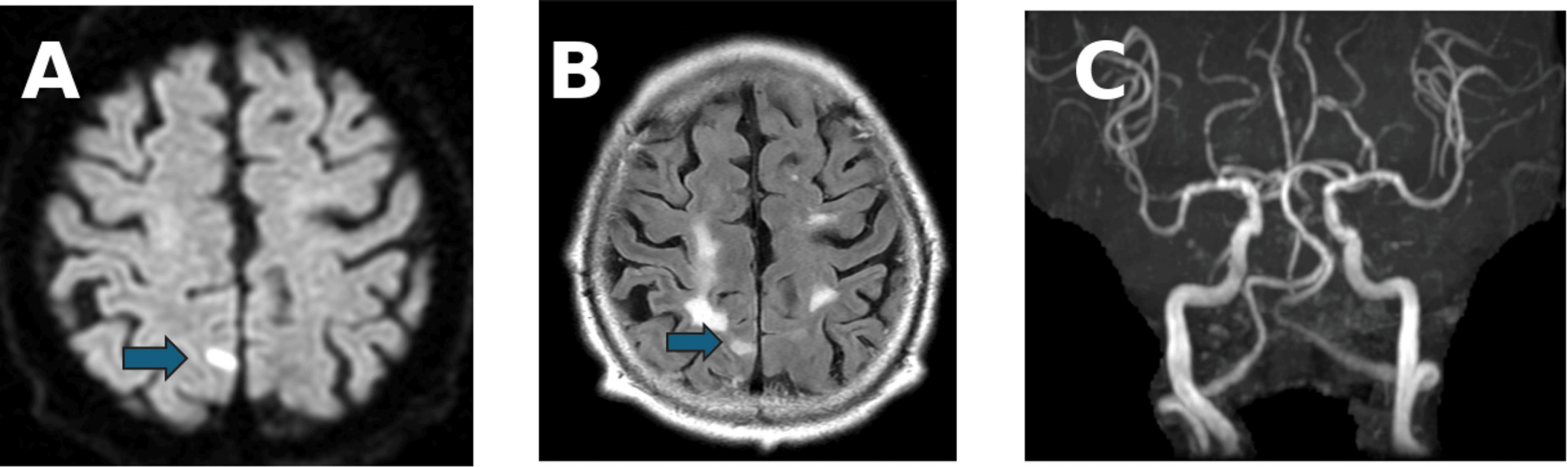
Brain MRI and angiographic findings. (A) Diffusion-weighted imaging demonstrating acute infarction in the right parietal lobe (arrow). (B) Fluid-attenuated inversion recovery imaging showing corresponding hyperintensity in the same region (arrow). (C) Magnetic resonance angiography revealing no significant large-vessel occlusion.

An 84-year-old man presented with left hemiparesis and impaired consciousness. On December 30 (year X-1), he fell at home and sustained a head injury. On January 1 (year X), he was transported to a previous hospital, where a head CT revealed no abnormalities, and he was discharged. On January 2, he developed sudden left-sided weakness and was transported to our hospital. MRI revealed an acute infarction in the right parietal lobe, and dual antiplatelet therapy was initiated (Figure 1).

**Figure 2:**
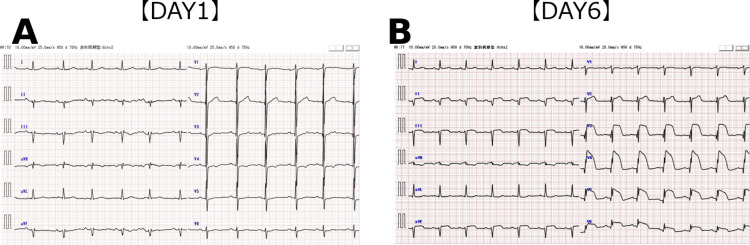
Electrocardiographic changes from admission to hospital day six. (A) Electrocardiogram on admission (day one) showing ST-segment abnormalities in the precordial leads. (B) Electrocardiogram on day six demonstrating dynamic changes with partial resolution of ST abnormalities.

On hospital day six, electrocardiography revealed diffuse ST-segment elevation without chest pain (Figure 2).

Transthoracic echocardiography demonstrated akinesis of the mid-to-apical segments of the left ventricle with compensatory hyperkinesis of the basal segments, resulting in a typical apical ballooning pattern. These findings were inconsistent with a single coronary artery distribution and supported the diagnosis of TTS.

**Figure 3:**
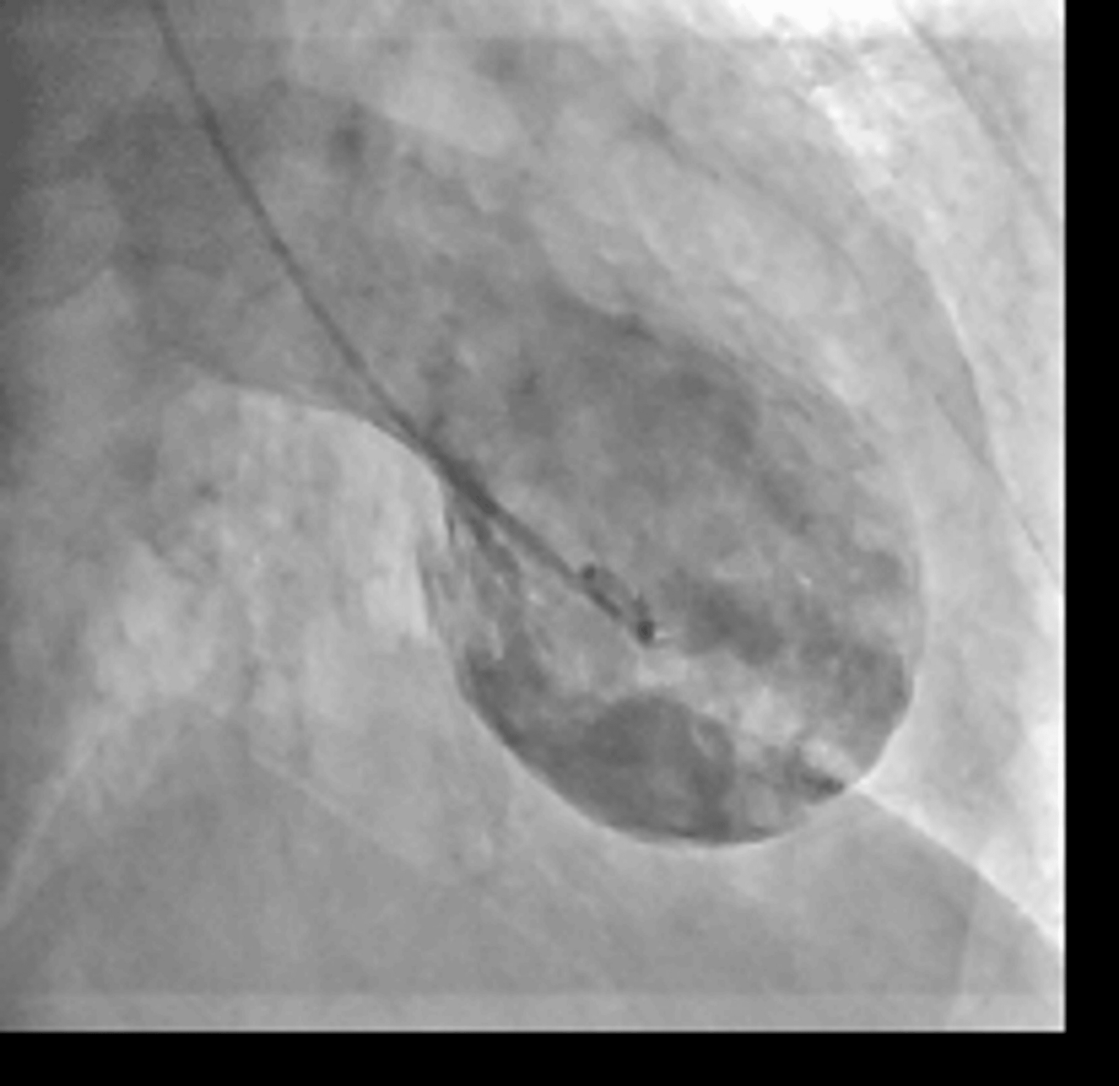
Left ventriculography showing apical ballooning. Left ventriculography demonstrated wall motion abnormalities extending beyond a single coronary artery territory, with akinesis of the mid-to-apical segments and basal hyperkinesis, consistent with Takotsubo syndrome.

Coronary angiography revealed approximately 75% stenosis in the proximal right coronary artery (segment #1) and approximately 75% stenosis in the left anterior descending artery (segment #6). These lesions were not considered flow-limiting. Left ventriculography demonstrated wall motion abnormalities that did not correspond to a single coronary artery territory (Figure 3).

Laboratory findings demonstrated elevation of cardiac biomarkers and inflammatory markers during the clinical course (Table 1).

**Table 1:** Temporal changes in laboratory findings with elevation of cardiac biomarkers. Laboratory findings showed a marked elevation of cardiac biomarkers, including troponin I and brain natriuretic peptide, on hospital day six compared to admission, indicating acute myocardial injury. These changes were accompanied by a mild increase in inflammatory markers, supporting the diagnosis of Takotsubo syndrome. WBC = white blood cell; CRP = C-reactive protein; CK = creatine kinase; CK-MB = creatine kinase-myocardial band; BNP = brain natriuretic peptide; BUN = blood urea nitrogen; PT-INR = prothrombin time-international normalized ratio; APTT = activated partial thromboplastin time

Parameter	Day 1	Day 6
WBC (/µL)	7,400	7,700
Hemoglobin (g/dL)	13.8	12.5
Platelets (/µL)	192,000	182,000
CRP (mg/dL)	0.115	2.155
Troponin I (ng/mL)	-	1.359
CK (U/L)	92	306
CK-MB (U/L)	-	23
BNP (pg/mL)	19.6	654
BUN (mg/dL)	17.9	13.4
Creatinine (mg/dL)	1.06	0.78
Sodium (mmol/L)	134	139
Potassium (mmol/L)	3.6	3.6
Chloride (mmol/L)	95	104
PT-INR	1	1.16
APTT (s)	22.6	29.4
D-dimer (µg/mL)	1	0.9

**Figure 4:**
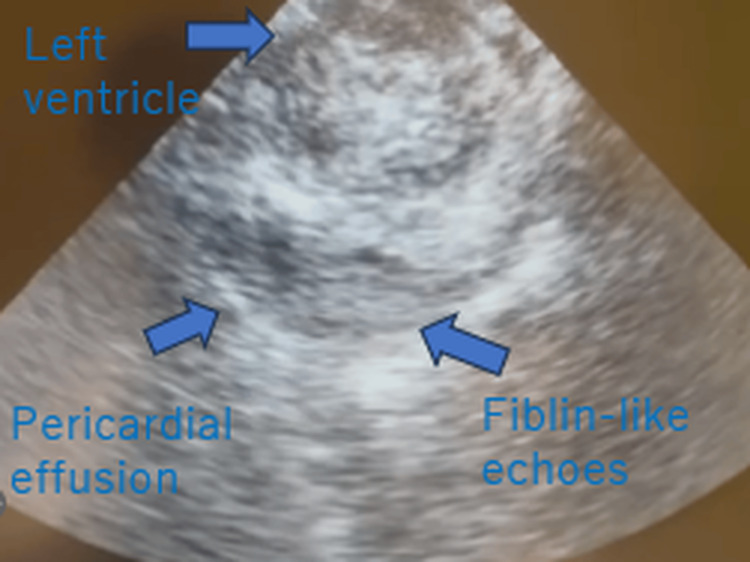
Transthoracic echocardiography showing pericardial effusion with fibrinous strands. Transthoracic echocardiography demonstrating pericardial effusion surrounding the left ventricle with echogenic fibrinous strands, consistent with organized hemopericardium associated with ventricular free wall rupture. Arrows indicate fibrinous echoes within the pericardial effusion adjacent to the left ventricular free wall.

Several hours later, the patient suddenly developed bradycardia followed by pulseless electrical activity and cardiac arrest. Echocardiography performed during resuscitation revealed pericardial effusion with fibrin-like echoes adjacent to the left ventricular free wall, strongly suggesting ventricular free wall rupture (Figure 4).

Despite resuscitative efforts, the patient died. The clinical course is summarized as follows: Day one: admission for acute ischemic stroke; dual antiplatelet therapy initiated. Day five: neurological status stable; rehabilitation started. Day six: asymptomatic diffuse ST-segment elevation → cardiology consultation. Several hours later: sudden bradycardia followed by collapse → cardiac arrest. During resuscitation: echocardiography revealed pericardial effusion with fibrinous echoes → suspected ventricular free wall rupture. Outcome: death despite resuscitative efforts.

## Discussion

This case highlights several important clinical aspects of TTS. First, acute ischemic stroke is a well-recognized trigger of TTS. Excessive sympathetic activation and catecholamine surge are thought to play a central role in myocardial stunning. Excess catecholamine exposure may also lead to microvascular dysfunction and increased wall stress, potentially contributing to myocardial fragility and subsequent rupture. Patients with neurological triggers have been reported to have worse outcomes [3].

Second, differentiation between TTS and ACS is often challenging, particularly in patients with coexisting coronary artery disease. In this case, coronary angiography revealed moderate stenoses (approximately 75% in the proximal right coronary artery and left anterior descending artery); however, the wall motion abnormalities observed on echocardiography and ventriculography extended beyond a single coronary territory. Additionally, the discrepancy between the degree of myocardial dysfunction and the relatively modest elevation of cardiac biomarkers compared to the extent of wall motion abnormalities supported the diagnosis of TTS rather than ACS.

Third, ventricular free wall rupture is an extremely rare but catastrophic complication. Previous reports suggest that rupture most commonly occurs in the left ventricular free wall and is associated with cardiac tamponade and sudden death, with a median time to rupture of approximately 48 hours [5]. Several factors have been proposed as potential risk indicators, including advanced age, persistent ST-segment elevation, elevated cardiac biomarkers, and hemodynamic instability. In the present case, progressive biomarker elevation and sudden hemodynamic deterioration may have indicated impending mechanical complications.

Compared with previously reported cases, rupture in TTS typically occurs within the first 48 hours. In the present case, rupture occurred within approximately 24 hours after onset, which is consistent with the reported time course, although relatively early.

Importantly, echocardiography played a pivotal role in this case. The detection of pericardial effusion and fibrin-like echoes adjacent to the left ventricular free wall strongly suggested ventricular free wall rupture. Echocardiography is a rapid and non-invasive modality that can be performed at the bedside and may be useful in identifying life-threatening mechanical complications.

However, the diagnosis of ventricular free wall rupture in this case remains presumptive, as no definitive confirmation, such as autopsy or surgical findings, was available. In addition, dynamic echocardiographic imaging and additional coronary imaging such as intravascular imaging were not available, which represent important limitations of this case.

Finally, the prognosis of TTS complicated by cardiac rupture is extremely poor, particularly in patients presenting with cardiac arrest, underscoring the importance of early recognition and close monitoring in high-risk patients.

## Conclusions

TTS is not always a benign condition and may be associated with fatal complications such as ventricular free wall rupture. Echocardiography may play an important role not only in the diagnosis of TTS but also in the early detection of life-threatening mechanical complications. In patients with neurological triggers such as acute ischemic stroke, careful monitoring is essential, as rapid clinical deterioration may occur. Early recognition and prompt assessment using echocardiography may aid clinical decision-making, although the prognosis remains poor once cardiac rupture develops.
